# Lactase persistence in Tunisia as a result of admixture with other Mediterranean populations

**DOI:** 10.1186/s12263-017-0573-3

**Published:** 2017-08-24

**Authors:** Yosra Ben Halima, Rym Kefi, Marco Sazzini, Cristina Giuliani, Sara De Fanti, Chokri Nouali, Majdi Nagara, Giacomo Mengozzi, Sahar Elouej, Abdelmajid Abid, Henda Jamoussi, Lotfi Chouchane, Giovanni Romeo, Sonia Abdelhak, Donata Luiselli

**Affiliations:** 1 Laboratory of Biomedical Genomics and Oncogenetics, Institut Pasteur de Tunis, BP 74, 13 Place Pasteur, 1002 Tunis, Tunisia; 2 Université de Tunis El Manar, 2092 El Manar I, Tunis, Tunisia; 3 Laboratory of Molecular Anthropology and Centre for Genome Biology, Department of Biological, Geological and Environmental Sciences (BiGeA), University of Bologna, 40126 Bologna, Italy; 4 Department of external consultation, National Institute of Nutrition and Food Technology, Tunis, Tunisia; 5 Laboratory of Genetic Medicine and Immunology, Weill Cornell Medical College in Qatar, Qatar Foundation, Doha, Qatar; 6 Medical Genetics Unit, Department of Medical and Surgical Sciences, Polyclinic S. Orsola-Malpighi, University of Bologna, Bologna, Italy

**Keywords:** Lactase persistence, North Africa, Admixture, Tunisia, Natural selection, *LCT*, *MCM6*

## Abstract

**Background:**

The ability to digest lactose after weaning, namely, lactase persistence (LP), is encoded by polymorphisms in the *MCM6* gene and varies widely in frequency among different human populations. Although, evolution of LP-related genetic variants was investigated in many groups of Sub-Saharan African, Middle Eastern, and European ancestry, only few studies have focused on populations from North Africa and no data are especially available from the Tunisian one. For this reason, there is an urgent need to investigate the frequency patterns at these loci in Tunisia since this adaptive trait is implicated in health.

**Methods:**

Forty SNPs covering the *LCT/MCM6* genes and including the two functional variants − 13,910 C > T and − 22,018 G > A were genotyped in 117 Tunisian individuals using the Sequenom Mass Array technology. The observed nucleotide and haplotype patterns of variation were then compared with those of several African, European, and Mediterranean human groups for which comparable data were publicly available. Admixture analysis on a 5 Mb genomic region surrounding the *LCT/MCM6* loci was also performed by extracting genotypes from a previously generated genome-wide dataset in order to deepen the reconstruction of the evolutionary history of these loci.

**Results:**

We found that lactase non-persistence (LNP)-related alleles and haplotypes were predominantly present in the examined population. A clear differentiation between Tunisian, African, and North European/North Italian samples was found, while the Tunisian population showed more genetic affinity to Central and South Italian groups.

**Conclusions:**

Our study provided a first report of LP-associated alleles and haplotypes in the Tunisian population. We highlighted a gradient followed by LP diffusion from Europe to North Africa. Based on the rich historic background of Tunisia, we suggest that this adaptive trait was introduced in that geographic region by a relatively recent gene flow.

**Electronic supplementary material:**

The online version of this article (doi:10.1186/s12263-017-0573-3) contains supplementary material, which is available to authorized users.

## Background

Lactase persistence (LP) or non-persistence (LNP) is a genetically determined trait related to the capacity of maintaining lactase activity till adulthood. LNP or lactase intolerance (LI) represents the ancestral state characterized by the downregulation of lactase activity [1]. Due to the continued expression of the *LCT* gene in some groups of North European descent, pastoralists from Africa, the Arabian Peninsula, and Central Asia [2], it is known that the lactase activity may persist even after weaning [2–7]. In fact, variants at the intronic regions in the neighboring *MCM6* gene were described to regulate transcriptional activity of the *LCT* promoter and to confer therefore LP [4].

Several reports from previous studies emphasized that the worldwide prevalence of the LP phenotype is highly variable between different ethnic groups and is positively correlated with the importance of milk in their diet. LP shows a highly structured global geographic distribution, it is indeed common in Europe, particularly in the northwestern regions [8], in parts of the Indian subcontinent and in many African and Middle Eastern populations that traditionally practice pastoralism and regularly consume milk [6, 9, 10]. It was thus hypothesized that LP evolved because it confers a selective advantage. For instance, the consumption of fresh milk and other dairy products is supposed to allow efficient caloric intake [11], calcium assimilation even at high latitude [12], and to increase water absorption from milk in arid environments [13].

Different LP-associated variants arose in several populations independently due to convergent evolution. In particular, the − 13,910 T (rs4988235) and − 22,018 A (rs182549) alleles were found to be 100 and 97% associated with LP, respectively, in the Finnish population [8]. Furthermore, the − 13,910 T allele is ~86–98% associated with LP in other European populations [14, 15]. However, in the Middle East and in most regions of Africa three other variants are commonly found in LP individuals (i.e., − 13,915 G, − 13,907 G, and − 14,010 C) [4, 16], as reported for instance for Ethiopian pastoralists [17]. Variation at LP-related loci was recently investigated in other groups from the South of Europe (i.e., Italy) and the Arabian Peninsula [18, 19], but not from the North of Africa. In fact, there are only few studies that reported the frequencies of LP-related alleles in Berber groups from Algeria and Morocco [20, 21].

Several hypotheses were suggested to explain the acquisition of the LP adaptive trait in North Africa. The first one is that the expansion of pastoralists from the Middle East into North Africa would presumably have resulted in the spread of lactose tolerance [11, 22]. The distribution of the − 13,910 T allele may thus provide useful answers concerning the origin of the spread of dairying [23]. Indeed, the age estimate for the − 13,910 T variant ranges from 12,300 to 5000 years ago [4, 20, 21, 24, 25], which broadly coincides with the origins of cattle domestication in North Africa and Levant around 10,000 years ago [26]. Indeed, the origin of the pastoral movement from the East and West of Africa to Tunisia was dated to the trading of gold, salt, and slaves across the Sahara [27]. Genetic studies conducted on populations from this area and performed using mitochondrial DNA (mtDNA) and nuclear markers confirmed also a correlation between genetic and geographic structure and/or distances [28] and a predominantly east-west structure [29]. Another hypothesis suggested by Myles and colleagues is that the − 13,910 T allele was introduced in North Africa from Europe via the Gibraltar Strait [30]. However, several studies showed that the Gibraltar Strait represented a strong boundary for gene flow [31–35]. In addition, archeological evidence indicates that the change to pastoralism in coastal North Africa was abrupt and not developed locally over a long period of time [36].

Tunisia has a geostrategic location since it represents a crossroad between Europe, the Middle East and Sub-Saharan Africa and a stepping-stone for recent human migrations. Therefore, the genetic background of the present Tunisian population may have been influenced by these migrations and the successive invasions of the country [27, 37]. The main focus of this study is thus to explore variation patterns of a large number of single nucleotide polymorphisms (SNPs) located in the *LCT/MCM6* region, potentially related to the LP phenotype. Accordingly, we compared newly generated data with those from worldwide human populations to test whether in Tunisia the same mutations as those found in the other parts of the world are observable and, finally, to determine if the distribution of allele frequencies underlying this trait are due to de novo-mutations or admixture with populations that were already characterized by LP (i.e., gene flow).

## Methods

### Sample collections and SNPs genotyping

The present study was carried out on 117 subjects collected from three different Tunisian geographic macro-areas: 61 samples from the North of Tunisia (NT), 29 samples from the center of Tunisia (CT), and 27 samples from the south of Tunisia (ST). The collection of blood samples was achieved with the collaboration of the National Institute of Nutrition (Tunis, Tunisia). The study was approved by the Ethics Committee of the Institut Pasteur de Tunis (Tunis, Tunisia-Registration numbers IRB00005445, FWA00010074), and all participants provided written informed consent.

DNA was extracted from blood samples using a salting out method, as described previously [38], and was used to genotype 40 informative SNPs selected over a wide genomic interval encompassing the *LCT/MCM6* loci and covering 3 Mb [18].

The Sequenom’s MassARRAY Designer software (Sequenom, Inc., San Diego, CA, USA) was used to design PCR and extension primers for the multiplex-PCR, with the total number of SNPs being divided in two multiplex. The first one was composed of 21 SNPs and the second one of 19 SNPs.

Genotyping was performed using the iPlex Gold Genotyping Assay and Sequenom MassArray DNA analysis [39] with Matrix-assisted laser desorption/ionization time-of-flight (MALDI-TOF) mass spectrometry (Sequenom, Inc., San Diego, CA, USA) at the Centre for Applied Biomedical Research (CRBA) of the Bologna S. Orsola University Hospital.

The obtained data for the Tunisian population (TN, *N* = 117) were compared with published datasets including 453 healthy Italian subjects [18] from North-Western and Central-Western Italy (NCWI, *N* = 105), North-Eastern Italy (NEI, *N* = 139), Central-Eastern and Southern Italy (CESI, *N* = 159), and from Sardinia (SARD, *N* = 47).Then, we merged these data with those for 10 populations of African and European ancestries from the 1000 Genomes Project [40]. Namely, 99 Utah residents, with North and West European ancestry (CEU), 99 Esan in Nigeria (ESN), 99 Finnish in Finland (FIN), 91 British in England and Scotland (GBR), 113 Gambian in Western Division (GWD), 107 Iberian populations in Spain (IBS), 99 Luhya in Webuye, Kenya (LWK), 85 Mende in Sierra Leone (MSL), 107 Tuscany in Italy (TSI), 108 Yoruba in Ibadan, Nigeria (YRI). The final merged dataset contained 38 common SNPs (one SNP was not found in the 1000 genomes data). Further information about the studied populations were reported in Additional file 1.

### Data analyses

#### Summary statistics and allele frequencies analyses

Summary statistics, such as nucleotide (π) and haplotype (H) diversity at the examined genomic regions and the number of haplotypes (*k*), were calculated for all the studied groups using the Arlequin package v.3.5.2 [41].

Allele frequencies for the common genotyped SNPs were calculated and compared between the examined population samples by applying a Chi-square test with the PLINK software package beta release 1.9 [42], significance level was set at 5%. Bonferroni’s correction was applied to the obtained asymptotic *p* values to account for the adopted multiple testing procedures.

#### Population structure and differentiation analyses

To test the genetic structure of our samples, we computed principal component analysis (PCA) by using the *adegenet* and *ade4* packages implemented in the R software [http://www.R-project.org/]. For this purpose, SNPs pairs that showed *r* ^2^ > 0.2 calculated on sliding window of 5 SNPs were removed from the dataset with PLINK by maintaining only one SNP per pair. After such a LD-pruning, 20 SNPs remained for the Tunisian dataset.

The Arlequin software was also used to calculate genetic diversity and pairwise Fst genetic distances between Tunisian samples and the other studied populations. Generated genetic distances (Fst matrix) were plotted to visualize data in multidimensional scaling (MDS) using the statistical package for the social science (SPSS, version 20.0, Chicago, IL, USA).

#### Discriminant analysis of principal components (DAPC)

To further corroborate results from population structure analyses, we evaluated the cluster membership probabilities for each subject by applying DAPC to the three identified Tunisian groups using the adegenet R package [43]. This approach allowed to provide assignment of individuals to different groups and to assess relationships between populations.

#### Admixture analyses

To better understand the genetic background and population structure of the LP-related genomic region, we extracted SNPs data for 5 Mb encompassing the *LCT* and *MCM6* loci and the surrounding genes from a dataset generated by means of the Genome-Wide Human SNP Array 6.0 (Affymetrix, Santa Clara, CA, USA) on 135 Tunisian healthy individuals that were recruited from the middle coast of Tunisia as described previously [44].

The study was conducted according to the declaration of Helsinki principles and approved by the Institutional review board (under the reference PV09/06, IRB#0000000044). These data were merged with the HapMap3 [45] and other publicly available data [46–48] to produce a dataset composed of 1560 variants for 1677 individuals (Additional file 1). The same quality control (QC) procedures used for filtering the Sequenom data (see Results section) were applied also to that dataset, which showed a mean genotyping rate of 77%. To avoid bias due to LD, variants that showed *r* ^2^ > 0.8 were filtered by pruning one SNP per five using the sliding windows approach implemented in the PLINK package and as a result 347 SNPs were removed.

Admixture proportions in the *LCT/MCM6* region were inferred for each subject included in the pruned dataset by means of the algorithm implemented in the software ADMIXTURE [49], which provides a maximum likelihood estimation of population structure. Individual genotypes were clustered by running an unsupervised analysis, testing numbers of potential ancestral populations (*K*) from two to eight, and by performing five iterations for each *K*.

#### Linkage disequilibrium and haplotype analyses

Patterns of LD at the genotyped loci were investigated using the software Haploview 4.2 [50]. Haplotypes were statistically inferred by considering all SNPs located within high LD blocks and through the Bayesian algorithm implemented in the PHASE software v.2.1 [51]. Evolutionary relationships of the reconstructed haplotypes were finally visualized by means of a median joining network [52] using the Network package v.4.6.1.1 [http://www.fluxus-engineering.com].

## Results

### Summary statistics of nucleotide and haplotype variation

Genotyping failed for four of the collected samples and individuals with more than 30% missing genotypes were excluded. One SNP (rs749017) for which experimental problems occurred during PCR reaction was also removed. The remaining 39 multiplexed SNPs showed call rates higher than 99% and minor allele frequencies (MAF) >5%. No loci showed a significant departure from Hardy-Weinberg equilibrium (HWE).

Allele frequencies for the typed functional SNPs −13,910 C/T and −22,018 G/A were calculated for all populations (Tables 1 and 2). The frequencies of reference alleles C and G and LNP associated genotype C/C were high in TN (Tables 1 and 2) and reached almost the same values in TSI, CESI, NEI, and IBS (Tables 1 and 2). The highest values were observed instead in ESN, GWD, LWK, MSL, and YRI (Tables 1 and 2), while the lowest ones were found, as expected, in Northern European populations, such as FIN, GBR, and CEU (Tables 1 and 2).

**Table 1:** Allelic frequencies for functional SNPs in the studied populations

Populations	rs4988235		rs182549	
	C (%)	T (%)	G (%)	A (%)
TN	88.07	11.92	85.89	14.10
NCWI	72.38	27.61	72.38	27.61
NEI	75.53	24.46	73.38	26.61
CESI	89.93	10.06	88.05	11.94
SARD	94.56	5.43	94.68	5.31
CEU	26.26	73.73	26.26	73.73
ESN	100	0	100	0
FIN	40.90	59.09	40.90	59.09
GBR	28.02	71.97	28.02	71.97
GWD	99.11	0.88	99.11	0.88
IBS	54.20	45.79	54.20	45.79
LWK	100	0	100	0
MSL	100	0	100	0
TSI	91.12	8.87	90.65	9.34
YRI	100	0	100	0

Allelic frequencies of functional SNPs in the studied populations are reported in Table 1. Frequencies for both reference and adaptive alleles were represented

**Table 2:** Genotypic frequencies for functional SNPs in the studied populations

Populations	rs4988235			rs182549		
	C/C (%)	C/T (%)	T/T (%)	G/G (%)	G/A (%)	A/A (%)
TN	77.98	20.18	1.83	73.50	24.78	1.70
NCWI	55.23	34.28	10.47	55.23	34.28	10.47
NEI	58.27	34.53	7.19	54.67	37.41	7.91
CESI	79.87	20.12	0	76.10	23.89	0
SARD	89.13	10.86	0	89.36	10.63	0
CEU	7.07	38.38	54.54	7.07	38.38	54.54
ESN	100	0	0	100	0	0
FIN	14.14	53.53	32.32	14.14	53.53	32.32
GBR	9.89	36.26	53.84	0	0	0
GWD	98.23	1.76	0	98.23	1.76	0
IBS	31.77	44.85	23.36	31.77	44.85	23.36
LWK	100	0	0	100	0	0
MSL	100	0	0	100	0	0
TSI	84.11	14.01	1.86	83.17	14.95	1.86
YRI	100	0	0	100	0	0

Genotypic frequencies of functional SNPs in the studied populations are reported in Table 2. Frequencies of different genotypes (C/C, C/T, and T/T) were represented

Since population structure analyses did not show appreciable differentiation patterns within TN (see next section), nucleotide diversity was calculated for the overall Tunisian population showing high values of diversity (0.400 ± 0.211) (Additional file 2: Table S1). We then compared Tunisian patterns of variation at the examined loci with those characterizing African and European populations. The obtained values for the TN population were comparable to those obtained for CESI, GBR, SARD, TSI, and CEU populations (0.399 ± 0.211, 0.392 ± 0.208, 0.390 ± 0.208, 0.366 ± 0.195, and 0.335 ± 0.180, respectively). In combination with the previously published data, Northern Italian groups, such as NEI and NCWI, showed the highest variability (0.472 ± 0.246 and 0.485 ± 0.252, respectively), with values similar to FIN and IBS (0.458 ± 0.239 and 0.489 ± 0.254, respectively). However, lower diversity was observed for ESN, GWD, LWK, YRI, and MSL (0.207 ± 0.119, 0.217 ± 0.124, 0.196 ± 0.114, 0.116 ± 0.075, and 0.167 ± 0.100, respectively). Summary statistics of nucleotide and haplotype variation for each group were reported in Additional file 2: Table S1.

### Population structure analyses

PCA was applied to the generated Tunisian genotypes and showed no clear genetic clustering of the different examined groups (Additional file 2: Figure S1). In addition, we performed DAPC that confirmed the absence of well-defined population clusters (Additional file 2: Figure S2).

**Fig. 1:**
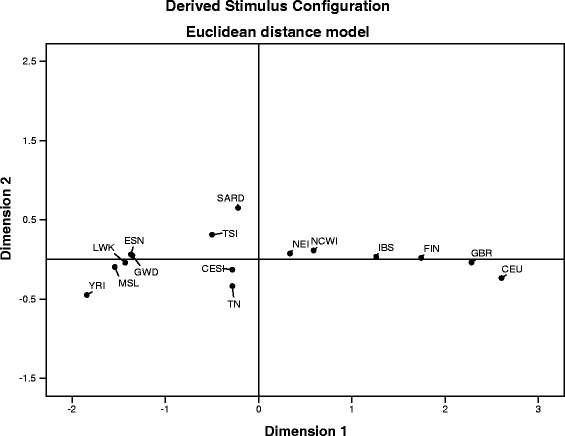
MDS of Fst estimates based on the LP region for several populations. Non-metric multidimensional scaling analysis of *LCT/MCM6* based on Fst distance showing the relationships among the studied populations. *ESN* Esan in Nigeria, *GWD* Gambian in Western Division, The Gambia, *LWK* Luhya in Webuye, Kenya, *MSL* Mende in Sierra Leone, *YRI* Yoruba in Ibadan, Nigeria, *TSI* Tuscany in Italy, *CESI* Central-Eastern Southern Italy, *NEI* North-Eastern Italy, *NCWI* North Central Western Italy, *SARD* Sardinia, *IBS* Iberian populations in Spain, *FIN* Finnish in Finland, *GBR* British in England and Scotland, *CEU* Utah residents (CEPH) with Northern and Western European ancestry, *TN* Tunisian population

Observed patterns of Tunisian variation were thus compared with those specific to African and European populations using Fst estimates computed for the 15 SNPs spanning the longest identified LD block (see LD block analysis, Additional file 2: Figure S3) and plotted using MDS (Fig. 1). Since PCA and DAPC applied to the Tunisian dataset showed no appreciable internal structure, the overall Tunisian population is considered as a single sample (TN). Accordingly, the first dimension differentiates mainly populations of African and European ancestry. TN was positioned near to the intersection of the axes in close proximity to the Southern European aggregate including CESI, TSI, and SARD, suggesting a higher European influence in TN than in the remaining African samples. African populations then appear to cluster in the lower left quadrant described by the second dimension.

In order to further explore patterns of population differentiation, allele frequencies for both functional SNPs were compared between TN and all populations by applying a Chi-square test (Additional file 2: Table S2). Tunisian groups were again pooled together since no significant results were obtained when considering the single subgroups. Unadjusted and adjusted differences corresponding to allelic frequencies for the typed SNPs are reported in Additional file 2: Table S2.

The most significant differences were observed mainly between TN and Northern European(i.e. CEU, GBR, and FIN) and Western European populations (i.e., IBS), followed by those compared to the African groups (i.e., GWD, YRI, MSL, ESN, and LWK). In contrast, the lowest differences were observed when we compared TN with Northern Italian samples (i.e., NEI and NCWI).Finally, no significant differences were found when Italian groups with a more Southern European ancestry (i.e., CESI, SARD, and TSI) were considered (Additional file 2: Table S2).

### Admixture analysis

**Fig. 2:**
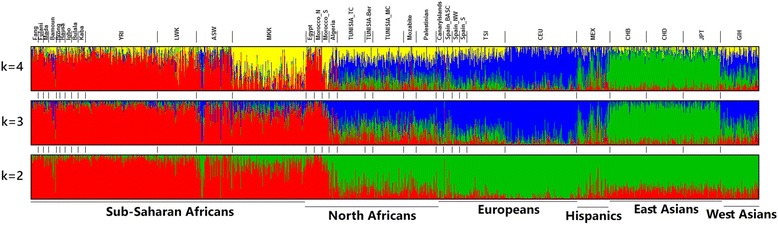
Ancestry proportions at *K* = 2 to *K* = 4 estimated by means of ADMIXTURE clustering analysis performed on the extended LP-related region. Population structure of worldwide human populations is shown for 1677 individuals from 33 different groups globally distributed (from left to right: 633 Sub-Saharan Africans, 225 North Africans, 399 Europeans, 77 Mexicans, 255 East Asians, 88 Indians)

We applied the unsupervised ancestry inference algorithm implemented in the ADMIXTURE software on the obtained pruned datasets. As the number of ancestral clusters increased, we observed the emergence of specific population clusters (Fig. 2). At *K* = 2, the ancestry assignment mainly differentiates an African-like (red) and a non-African-like (green) component. *K* = 3 further distinguishes an Asian-like (green) ancestry fraction from a European-like one (blue), while *K* = 4 identifies an additional component (yellow) that is mainly shared among East, Sub-Saharan, and North African clusters. Remarkably, the Tunisian population, and at a lower extent, the Algerian samples appeared to be clearly differentiated from the other African populations. They showed more than 95% contribution from different ancestral populations along different *K* values. When using the cross validation error, the mean log probability for the successive increase of *K* levels continues to decrease substantially as *K* increases. Although, higher values of *K* reveal additional population-specific ancestries until *K* = 4. For this reason, we focused on *K* = 2 through *K* = 4.

### LD block analysis

The analysis of LD patterns that was carried out on the Tunisian dataset showed the existence of a long block of relatively high linkage disequilibrium (266 kb), which includes the two functional variants − 13,910 C/T and −22,810 G/A (Additional file 2: Figure S3a). Indeed, the NT group presented a slightly shorter block (157 kb) that contains only six SNPs of the 15 variants observed when the total population was considered (Additional file 2: Figure S3b). However, in CT, the same long haplotype (266 kb) was observed and includes a different SNP (rs1438307) (Additional file 2: Figure S3c). Interestingly, this region of high LD appeared to be split into two different blocks spanning respectively (12 kb) and (185 kb) in the ST group (Additional file 2: Figure S3d).

### Haplotype reconstruction

We statistically inferred the haplotypes in all the samples that belong to the Tunisian population by considering the 15 SNPs located in the region of overall high LD that is shared among the examined NT, CT, and ST groups. We thus observed a total of 23 different haplotypes, 21 of which carried the − 13,910 C allele and two of them (H19 and H23) showed the functional allele − 13,910 T (Additional file 2: Table S3). The most frequent haplotype (H1) carried the − 13,910 C and the −22,080 G reference alleles, being remarkably represented in NT, CT, and ST groups (0.57, 0.64, and 0.54, respectively). The second most frequent haplotype (H19) carried instead the adaptive alleles, being mainly represented in NT (0.14) and showing instead lower frequencies in CT and ST samples (0.10 and 0.10, respectively).

**Fig. 3:**
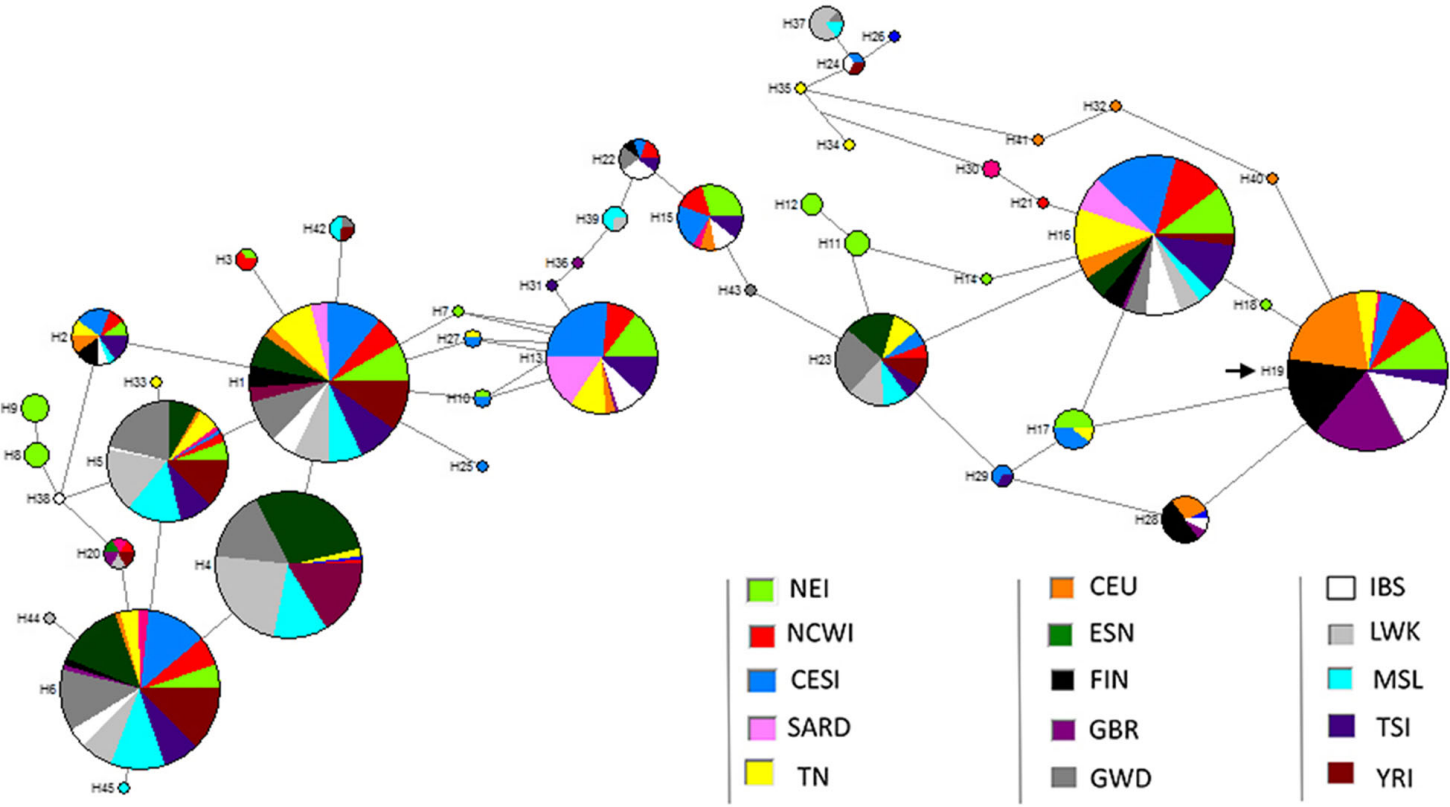
Median joining network of the inferred haplotypes. Haplotypes proportions were displayed with different colors according to the different examined populations. Adaptive haplotype was indicated by arrow

When we compared the Tunisian population to the other populations, we found that it presented several private haplotypes (e.g., H33, H34, and H35) that carry the reference − 13,910 C and − 22,080 G alleles. The most frequent haplotype (H1) in the merged dataset (Additional file 2: Table S4) carried the − 13,910 C and the − 22,080 G alleles and was represented in all populations. It reaches the highest frequencies in YRI, TN, TSI, MSL, GWD, LWK, CESI, SARD, NEI, ESN, and NCWI, (0.66, 0.58, 0.57, 0.56, 0.56, 0.53, 0.53, 0.51, 0.44, 0.49, and 0.41, respectively). In contrast, the second most common haplotype (H19)which carries the adaptive alleles − 13,910 T and − 22,080 A was highly represented in GBR, CEU, and FIN (0.71,0.70, and 0.55, respectively) and absent in ESN, LWK, MSL, and YRI. The remaining haplotypes finally appeared to be rare and, in some cases, private. Evolutionary relationships among the haplotypes inferred in the studied populations were further explored using a median joining network that highlighted the presence of two clearly distinct groups of allelic combinations separated by several mutational steps. Thus, the haplotype carrying the derived alleles (H19) was segregated into a single cluster and was separated from those carrying the ancestral alleles (H1) (Fig. 3).

## Discussion

In the present study, we reported the frequencies and genotypes of the − 13,910 C/T and − 22,810 G/A functional SNPs related to LP [18]. We further described the distribution patterns of *LCT/MCM6* haplotypes in the Tunisian population, and we compared them to those observed in several groups of African and European ancestry that could have intervened in the formation of the Tunisian genetic background as recently hypothesized [48].

The obtained results were discussed in the light of the relatively recent historical relationships between Tunisian and the abovementioned populations [27, 53, 54], in the attempt to clarify the routes followed by LP diffusion and to explore the potential processes of convergent evolution of LP in European and in some non-European populations, such as the Tunisian one.

Investigation of the impact of admixture on a large genomic region related to LP showed that the Tunisian population is highly mixed and displays three major ancestral contributions ascribable to European, African, and Asian ancestry components (Fig. 2). This is in agreement with previous studies that examined uniparental and autosomal markers and showed a multi-ethnic origin of North Africans [27, 37, 46].

Moreover, the study of LP-related genetic diversity revealed that even within the relatively small region represented by Tunisia, a high nucleotide diversity (0.400 ± 0.212) at *MCM6/LCT* region could be observed (Additional file 2: Table S1). Many studies demonstrated that diversity varied substantially within and between Tunisian and other populations [27, 54]. Furthermore, according to our admixture analysis, the Tunisian population showed higher European proportion compared to other African groups. This was expected if we consider the roots of human migration in Africa. These results corroborate findings from several recent studies [46, 55] and suggest that the TN population has plausibly acquired the LP adaptive variants mainly through admixture with populations of European ancestry.

Our study highlighted an important prevalence of alleles and genotypes related to LI in the Tunisian population, as well as in South European and African groups (Tables 1 and 2). As expected, we found that the North European populations showed the lowest frequencies for these alleles and these findings were consistent with what was found previously [8, 18, 56, 57]. Furthermore, the allele frequencies of the typed SNPs exhibited great variation between Tunisians versus North European and North Italian groups (Additional file 2: Table S2). On the contrary, no differences were detected when we compared different groups from the Mediterranean regions. This distribution of LP-associated − 13,910 T and − 22,018 A alleles is in agreement with the north-south decreasing pattern observed by Anagnostou et al. [58] and De Fanti et al. [18] along the Italian peninsula.

The present study further highlighted the close affinity of the Tunisian population to the South European and Mediterranean populations than to African ones if we consider the LP-related genetic variants. Our data thus support the hypothesis that this adaptive trait was introduced to Tunisia by a relatively recent gene flow and did not evolve locally and independently as occurred for the bulk of Sub-Saharan African groups.

To draw a first picture of the potentially LP-related patterns in the Tunisian population, LD analysis (Additional file 2: Figure S3a, b, c, and d) was also carried out. The presence of a long haplotype block can be interpreted as evidence of a more recent introduction of LP especially in the CT subgroup with respect to those of NT and ST. Our data showed that the − 22,810 A allele was in strong LD with the LP-associated − 13,910 T variant in TN, which is in agreement with prior studies conducted on European populations [8, 18, 24] and to the hypothesis that positive selection at the − 13,910 T allele led to the concomitant rise in frequency of other polymorphisms near to the functional SNP determining the creation of a conserved haplotype block, as observed by Bersaglieri et al. [24].

Finally, we investigated the patterns of Tunisian haplotype variation. We found that the haplotype (H1) carrying the reference LI alleles was the most frequent in all the Tunisian and Italian subgroups, as well as in most African populations. However, the second most frequent haplotype (H19) that carries the adaptive alleles showed the highest frequencies in Northern European populations (i.e., FIN, GBR, and CEU) (Additional file 2: Table S4). Our findings suggest that the distribution of the observed haplotype variation might be due to gene flow that occurred over time from outside or within Africa possibly during key historical events, such as the settlement of the Roman Empire in parts of North Africa. These migration events are also reported in studies based on mtDNA, Y chromosome, and autosomal genetic variation [7, 27, 31, 59, 60]. On the other hand, population structure analyses, such as the MDS plot (Fig. 1), showed that the Tunisian sample was relatively close to the Italian groups. Furthermore, it is differentiated from African populations and especially clustered with samples from Central-Eastern and Southern Italy, supporting the occurrence of appreciable gene flow from Southern Europe to North Africa.

Accordingly, the present study provided a very first view of the genetic structure of the Tunisian population considering the genomic region that modulates the LP phenotype by analyzing subpopulations distributed geographically along the Tunisian territory. Moreover, the investigation of diversity at a wide genomic interval surrounding the *LCT* gene could potentially help to detect genetic variants that may contribute to the development of certain diseases [61]. Indeed, LP was previously shown to be able to confer susceptibility to certain diseases, such as obesity or metabolic disorders [62]. The LP alleles may thus contribute to the complexity of the currently novel and obesogenic environment also in Tunisia.

## Conclusions

Our study was the first to report the distribution of LP-associated alleles and haplotypes in the Tunisian population. We thus described the gradient followed by LP diffusion from Europe to Northern Africa. Based on the rich history of gene flow documented for this population, we support the hypothesis of introgression of the LP-associated alleles from outside the region. For this reason, we suppose that the current patterns of diversity observed at this locus in Tunisia may be the result of the interaction of a large number of evolutionary factors, such as genetic drift, demographic processes, people migrations, and admixture.

Further molecular investigation and dissection of the underlying evolutionary forces are needed to fully understand this phenomenon. It could be possible in the future to identify other loci than those on the *LCT/MCM6* that have undergone recent positive selection and that contributed to the modulation of the LP phenotype, thus leading to new insights into human evolution.

## Additional files

Additional file 1: Supplementary information about the studied populations. (ZIP 22 kb)

Additional file 2: Figure S1. Principal component analysis (PCA) plot of genetic variation in Tunisian groups. Distribution of northern, central, and southern samples in the space of two first component of LP-related genotypes. **Figure S2.** Discriminant analysis of principal component (DAPC) in Tunisian groups. DAPC analysis confirmed results of PCA. **Figure S3.** Linkage disequilibrium patterns (LD) of the investigated genomic region in Tunisian population. Each number in squares indicates the *r* ^2^ index of LD between the corresponding SNPs. **a** Tunisian population. b Northern Tunisian group. c Central Tunisian group. d Southern Tunisian group. **Table S1.** Summary statistics for the studied populations. Table includes number of chromosome, nucleotide diversity, number of haplotypes, and haplotype diversity. **Table S2.** Results of allelic frequencies comparison between Tunisia and African and European populations. A chi-square test was used to test allelic differences; to reduce the false discovery rate of multiple testing, Bonferroni’s multiple comparison adjustment was performed. The statistical significance reached a *p* value less or equal to 5%. **Table S3.** Haplotype list in Tunisian population. A list of 23 different haplotypes generated by PHASE software, distributed between Northern, Central, and Southern Tunisian subgroups. **Table S4.** Haplotype list in the merged dataset. A list of 45 different haplotypes generated when comparing our data with other populations. (ZIP 608 kb)
