# Supplementary material for: Lactase persistence in Tunisia as a result of admixture with other Mediterranean populations
Source: Genes Nutr. 2017 Aug 24;12:20. doi: 10.1186/s12263-017-0573-3 (PMC5571577; doi:10.1186/s12263-017-0573-3)
Supplement: Supplementary file 1 — Supplementary information about the studied populations. (ZIP 22 kb) [file 12263_2017_573_MOESM1_ESM.zip › Table-S2_Supplementary informations of the worldwide Human population.docx]

Table S2: Genotyping data for worldwide Human populations (dataset2)

| Population | Population code | Sample size | Country | References |
| --- | --- | --- | --- | --- |
| **Tunisian New sampling**  **(135 samples)** | TUNISIA_MC | 71 | Tunisia | Our study |
|  | TUNISIA_TC | 64 |  |  |
| **North African data (119 samples)** | TUNISIA_berbers | 18 | Tunisia | Brenna M. Henn et al. 2012 |
|  | Morocco - North | 18 | Morocco |  |
|  | Morocco - South | 16 | Morocco |  |
|  | Algerian | 19 | Algeria |  |
|  | Mozabite | 29 | Algeria |  |
|  | Egyptian | 19 | Egypt |  |
| **European data**  **(324 samples)** | Spain_Basc | 20 | Spain |  |
|  | Spain_NW | 17 | Spain |  |
|  | Spain_S | 17 | Spain |  |
|  | CanaryIslands | 17 | Spain |  |
|  | CEU | 165 | Northern and Western European | HapMap project database |
|  | HapMap-TSI | 88 | Toscans-Italy |  |
| **Western and central Africa (633 samples)** | Bulala | 15 | Nigeria | Brenna M. Henn et al. 2012 |
|  | Fulani | 12 | Nigeria |  |
|  | Mada | 12 | Madagascar |  |
|  | Kaba | 17 | Central African |  |
|  | Fang | 15 | Gabon |  |
|  | Bamoun | 18 | Cameroon |  |
|  | Brong | 8 | Ghana |  |
|  | Hausa | 12 | Cameroon |  |
|  | Igbo | 15 | Nigeria |  |
|  | HapMap-YRI | 166 | Nigeria | HapMap project |
|  | HapMap-ASW | 83 | African ancestry-Southwest USA |  |
|  | HapMap-LWK | 90 | Luhya-Kenya |  |
|  | HapMap-MKK | 170 | Maasai-Kenya |  |
| **Middle Eastern , Hispanics and**  **Asian populations**  **(466 samples)** | HapMap-MEX | 77 | Mexican-Los Angelos |  |
|  | HapMap-CHB | 84 | China |  |
|  | HapMap-CHD | 85 | China |  |
|  | HapMap-JPT | 86 | Japan |  |
|  | HapMap-GIH | 88 | India-Houston |  |
|  | Palestinian | 46 | Middle East | Brenna M. Henn et al. 2012 |
