# Supplementary material for: Lactase persistence in Tunisia as a result of admixture with other Mediterranean populations
Source: Genes Nutr. 2017 Aug 24;12:20. doi: 10.1186/s12263-017-0573-3 (PMC5571577; doi:10.1186/s12263-017-0573-3)
Supplement: Supplementary file 1 — Supplementary information about the studied populations. (ZIP 22 kb) [file 12263_2017_573_MOESM1_ESM.zip › Table_S1_Supplementary informations of the studied populations.docx]

Table S1: Genotyping data for studied populations (dataset1)

| Population code | Population | Sample size | Reference |
| --- | --- | --- | --- |
| NT | Northern Tunisia | 61 | The present study |
| CT | Central Tunisia | 29 | The present study |
| ST | Southern Tunisia | 27 | The present study |
| NCWI | North Western and Central Western Italy | 105 | De Fanti et al.2015 |
| NEI | North Eastern Italy | 139 | De Fanti et al.2015 |
| CESI | Central Eastern and Southern Italy | 159 | De Fanti et al.2015 |
| SARD | Sardinia | 47 | De Fanti et al.2015 |
| CEU | Utah residents (CEPH) with Northern and Western European ancestry | 99 | 1000 Genomes Project database |
| ESN | Esan in Nigeria | 99 | 1000 Genomes Project database |
| FIN | Finnish in Finland | 99 | 1000 Genomes Project database |
| GBR | British in England and Scotland | 91 | 1000 Genomes Project database |
| GWD | Gambian in Western Division, The Gambia | 113 | 1000 Genomes Project database |
| IBS | Iberian populations in Spain | 107 | 1000 Genomes Project database |
| LWK | Luhya in Webuye, Kenya | 99 | 1000 Genomes Project database |
| MSL | Mende in Sierra Leone | 85 | 1000 Genomes Project database |
| TSI | Toscani in Italy | 107 | 1000 Genomes Project database |
| YRI | Yoruba in Ibadan, Nigeria | 108 | 1000 Genomes Project database |
