# Supplementary figures and images for: Lactase persistence in Tunisia as a result of admixture with other Mediterranean populations
Source: Genes Nutr. 2017 Aug 24;12:20. doi: 10.1186/s12263-017-0573-3 (PMC5571577; doi:10.1186/s12263-017-0573-3)

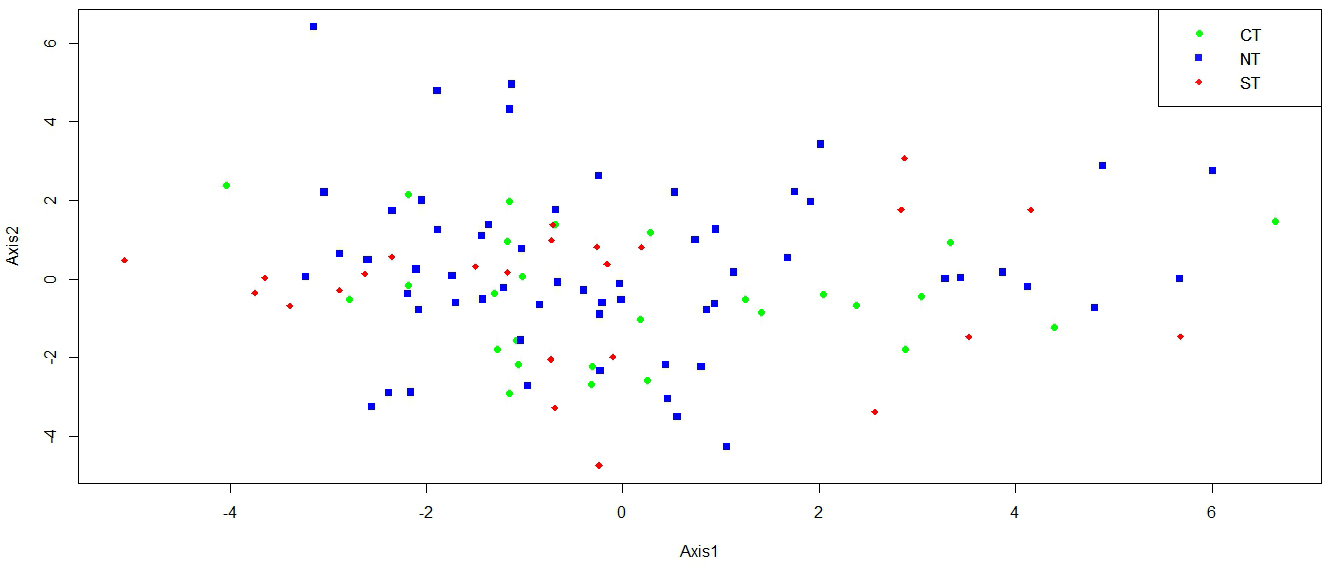

Supplement: Supplementary file 2 — Principal component analysis (PCA) plot of genetic variation in Tunisian groups. Distribution of northern, central, and southern samples in the space of two first component of LP-related genotypes. Figure S2. Discriminant analysis of principal component (DAPC) in Tunisian groups. DAPC analysis confirmed results of PCA. Figure S3. Linkage disequilibrium patterns (LD) of the investigated genomic region in Tunisian population. Each number in squares indicates the r 2 index of LD between the corresponding SNPs. a Tunisian population. b Northern Tunisian group. c Central Tunisian group. d Southern Tunisian group. Table S1. Summary statistics for the studied populations. Table includes number of chromosome, nucleotide diversity, number of haplotypes, and haplotype diversity. Table S2. Results of allelic frequencies comparison between Tunisia and African and European populations. A chi-square test was used to test allelic differences; to reduce the false discovery rate of multiple testing, Bonferroni’s multiple comparison adjustment was performed. The statistical significance reached a p value less or equal to 5%. Table S3. Haplotype list in Tunisian population. A list of 23 different haplotypes generated by PHASE software, distributed between Northern, Central, and Southern Tunisian subgroups. Table S4. Haplotype list in the merged dataset. A list of 45 different haplotypes generated when comparing our data with other populations. (ZIP 608 kb) [file 12263_2017_573_MOESM2_ESM.zip › Figure_S1_PCA plot of genetic variation in Tunisian population.jpg]

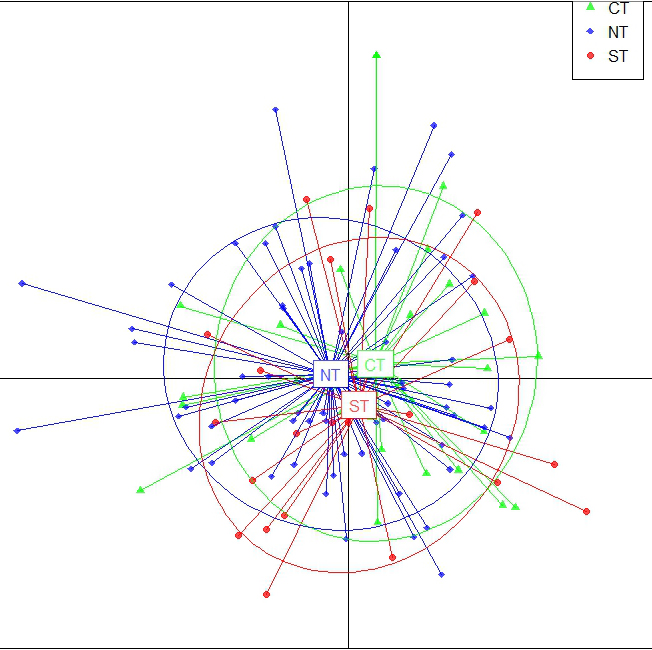

Supplement: Supplementary file 2 — Principal component analysis (PCA) plot of genetic variation in Tunisian groups. Distribution of northern, central, and southern samples in the space of two first component of LP-related genotypes. Figure S2. Discriminant analysis of principal component (DAPC) in Tunisian groups. DAPC analysis confirmed results of PCA. Figure S3. Linkage disequilibrium patterns (LD) of the investigated genomic region in Tunisian population. Each number in squares indicates the r 2 index of LD between the corresponding SNPs. a Tunisian population. b Northern Tunisian group. c Central Tunisian group. d Southern Tunisian group. Table S1. Summary statistics for the studied populations. Table includes number of chromosome, nucleotide diversity, number of haplotypes, and haplotype diversity. Table S2. Results of allelic frequencies comparison between Tunisia and African and European populations. A chi-square test was used to test allelic differences; to reduce the false discovery rate of multiple testing, Bonferroni’s multiple comparison adjustment was performed. The statistical significance reached a p value less or equal to 5%. Table S3. Haplotype list in Tunisian population. A list of 23 different haplotypes generated by PHASE software, distributed between Northern, Central, and Southern Tunisian subgroups. Table S4. Haplotype list in the merged dataset. A list of 45 different haplotypes generated when comparing our data with other populations. (ZIP 608 kb) [file 12263_2017_573_MOESM2_ESM.zip › Figure_S2_DAPC analysis in Tunisian groups.jpg]
